# PARTAKE Survey of Public Knowledge and Perceptions of Clinical Research in India

**DOI:** 10.1371/journal.pone.0068666

**Published:** 2013-07-16

**Authors:** Tal Burt, Savita Dhillon, Pooja Sharma, Danish Khan, Deepa MV, Sazid Alam, Sarika Jain, Bhavana Alapati, Sanjay Mittal, Padam Singh

**Affiliations:** 1 Duke Global Proof-of-Concept (POC) Research Network, Duke Clinical Research Unit (DCRU) & Duke Clinical Research Institute (DCRI), Duke University, Durham, North Carolina, United States of America; 2 Department of Psychiatry and Behavioral Sciences, Duke University, Durham, North Carolina, United States of America; 3 Medanta Duke Research Institute (MDRI), Gurgaon, India; 4 Medanta the Medicity, Medanta Institute of Education and Research (MIER), Gurgaon, India; 5 Medanta Institute of Education and Research (MIER), Gurgaon, India; 6 Clinical Cardiology & Research, Medanta -The Medicity Hospital, Gurgaon, India; The George Washington University Medical Center, United States of America

## Abstract

**Background:**

A public that is an informed partner in clinical research is important for ethical, methodological, and operational reasons. There are indications that the public is unaware or misinformed, and not sufficiently engaged in clinical research but studies on the topic are lacking. PARTAKE – Public Awareness of Research for Therapeutic Advancements through Knowledge and Empowerment is a program aimed at increasing public awareness and partnership in clinical research. The PARTAKE Survey is a component of the program.

**Objective:**

To study public knowledge and perceptions of clinical research.

**Methods:**

A 40-item questionnaire combining multiple-choice and open-ended questions was administered to 175 English- or Hindi-speaking individuals in 8 public locations representing various socioeconomic strata in New Delhi, India.

**Results:**

Interviewees were 18–84 old (mean: 39.6, SD±16.6), 23.6% female, 68.6% employed, 7.3% illiterate, 26.3% had heard of research, 2.9% had participated and 58.9% expressed willingness to participate in clinical research. The following perceptions were reported (% true/% false/% not aware): ‘research benefits society’ (94.1%/3.5%/2.3%), ‘the government protects against unethical clinical research’ (56.7%/26.3%/16.9%), ‘research hospitals provide better care’ (67.2%/8.7%/23.9%), ‘confidentiality is adequately protected’ (54.1%/12.3%/33.5%), ‘participation in research is voluntary’ (85.3%/5.8%/8.7%); ‘participants treated like ‘guinea pigs’’ (20.7%/53.2%/26.0%), and ‘compensation for participation is adequate’ (24.7%/12.9%/62.3%).

**Conclusions:**

Results suggest the Indian public is aware of some key features of clinical research (e.g., purpose, value, voluntary nature of participation), and supports clinical research in general but is unaware of other key features (e.g., compensation, confidentiality, protection of human participants) and exhibits some distrust in the conduct and reporting of clinical trials. Larger, cross-cultural surveys are required to inform educational programs addressing these issues.

## Introduction

**Figure 1:**
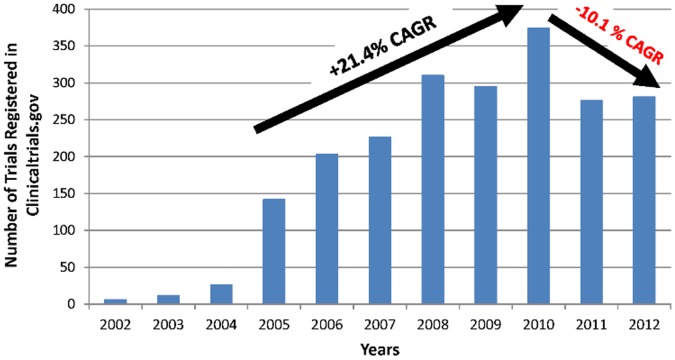
India clinical trials (all types) registered in Clinicaltrials.gov (2002–2012); Compound Annual Growth Rate (CAGR). Compound Annual Growth Rate (CAGR) was used for the periods 2005–2010 and 2010–2012 as follows: 
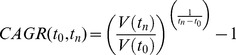
 V(t_0_) – start value; V(t_n_) – end value; t_n_ – t_0_ : number of years.

The promise of independent, indigenous medical research and new therapeutic development is not only attractive but indispensable for emerging economies as India, poised to be the world’s most populous and a leading economy by the mid-21^st^ century [1]. Indian clinical trial sector and involvement in international collaborations are expected to rise [2]. Currently, however, challenges in execution of clinical trials in India lead to dependence on extrapolation of Western data as the primary source of innovative therapeutics in the country. Unlike the West, clinical research represents a new venture for the Indian society and the national economy. The country that represents 17.5% of the world population still accounts for only 1.4% of global clinical research (Clinicaltrials.gov, 2012 [calculated for the period August 7, 2011– August 6, 2012]) [3]–[5]. In addition, recently, there has been a decline in the number of clinical trials conducted in India with a negative 10.1% Compound Annual Growth Rate (CAGR) for the 2010–2012 period after a positive 21.4% CAGR for the 2005–2010 period (Source: Clinicaltrials.gov; Figure. 1).

Public awareness, perceptions and consequent attitudes towards clinical research may impact regulatory policies, guide research priorities, and shape growth in the sector. Distrust, lack of awareness, and misconceptions of clinical research have been identified as key barriers to participation in clinical trials [6]–[10].

Patients, their families, caregivers, advocacy groups, and the public at large are integral to medical research process. They should be kept informed not only of the specific protocols they contribute to but also of the entire clinical research process for the following reasons:

1. Ethical reasons: these can be divided into rights and obligations:
  - Rights: Participants in clinical research have the right to make informed decisions about participation in research and, if choosing to participate, have the rights to autonomy, justice, beneficence, confidentiality, and adequate compensation for adverse outcomes [11]–[13]. Participants in research have the right to have their preferences and values respected [14]. Finally, informed individuals are better positioned to protect their rights [15], [16].
  - Obligations: Societies who desire and demand advanced therapeutics and individuals who are willing recipients of innovative treatments – imply an obligation to be part of the process that develops and approves them. Consumers of healthcare have the responsibility to participate in the research that informs care decisions [14].
2. Methodological reasons**:** a wide and representative sample of participants in clinical research is essential to ensure adequate generalization of the findings to the population at large [17], [18]. Informed public and research participants could assist in enforcement of research standards.
3. Operational reasons: a key obstacle to medical progress is the limited availability of human volunteers (both healthy and patients) for participation in clinical research. This makes research more costly, less powerful in detecting meaningful therapeutic effects, and delays the arrival of new treatments to those who need them [10], [18]–[21].

### Enforcement of Ethical Standards

Both professional and lay press has expressed concerns about the vulnerability of the Indian population, especially the illiterate and the poor, to exploitation, misinformation, and lack of information [22]–[24]. Risk-averse and resource-limited government and regulators have responded by increasing oversight and length of time required for approval of clinical research protocols [4], [25]. Likewise, risk-averse industry sponsors may hesitate to conduct clinical research in environments prone to ethical irregularities. Even local sponsors are considering taking their business outside of the country. These dynamics may be associated with the observed reversal of the growth trend in the sector (Figure 1).

### Negative Reports in the Media and Professional Press

Notwithstanding the important role of the media in disseminating information about unethical practices, unflattering and incorrect depictions of clinical research in the media persist [26]. Such reports have the potential to generate an ‘exploitative’ image [27] of research sponsors, and lead to distrust, lack of support and opposition to clinical research [6]–[9]. Following are a few examples. Medindia.com, a website that describes itself as “Asia’s premier health portal” has the following quote in one of the articles: “Due to intensive and strict Animal guidelines using animals in India too has become a very [sic] problem, so the drug companies have shifted their trials to humans rather [sic] to animals” [28]. After four teenage girls taking part in the Human Papilloma Virus (HPV) vaccine died a formal investigation into the matter found the reasons for the deaths unrelated to the vaccines, however, the Indian government is still being accused in the media of allowing the public to be used as ‘guinea pigs’ to test dangerous vaccines [29]. A review of public opinion polls [30] reported that 39% thought pharmaceutical companies failed to serve consumers (higher than 19% in 1997) but failed to mention that 60% thought the pharmaceutical companies did a good job serving their consumers (higher than 44% in 2004) [31].

### Studying and Enhancing Public Awareness of Clinical Research in India

Available data on public awareness, knowledge and perceptions of clinical research in India are limited and qualitative in nature and cover mostly attitudes about participation as volunteers in clinical trials [25], [32], [33], not the wider scope of engagement in clinical research policies, advocacy, sponsoring and partnership [34]. A meta-analysis of 7 studies (4 in India) of factors associated with participation in clinical research has identified the following factors as barriers to participation among members of the public [32]: Mistrust of trial organizations –26%, concerns about efficacy and safety of trials –21%, dependency issues (need to obtain approval for participation in research from another individual) –19%, loss of confidentiality –17%, trial burden –11%, psychological reasons –6%, and language –1%. Of these concerns, arguably the majority (mistrust of trial organizations, concerns about safety and efficacy, and loss of confidentiality, for a total of 64%) are amenable to preventive education and clarifications, reassurance, and guarantees by trial operators and related institutions (e.g., investigators, regulatory authorities).

Public awareness and advocacy campaigns have been shown to produce meaningful increase in awareness and participation in clinical research [18], [19]. The PARTAKE program (Public Awareness of Research for Therapeutic Advancements through Knowledge and Empowerment) was created to study gaps in awareness and extent of misinformation and inform corrective educational programs [21], [35]. This report describes one of its elements – the PARTAKE Survey of Knowledge and Perceptions of Clinical Research.

## Methodology

### 

#### 1. Clinicaltrials.gov methodology

(Data were obtained from Clinicaltrial.gov on February 16th, 2013 [3]. Methods: ‘Advanced Search’ option was used. ‘India’ entered in ‘Country’ field’. ‘First Received’ field was used to include dates ‘From ‘01/01/….’ To ‘12/31/….’, for each year from 2002 through 2012. The following Compound Annual Growth Rate (CAGR) was used for the periods 2005–2010 and 2010–2012 (years prior to 2005 were deemed to contain data that was not meaningful because the number of studies was very low [<30 per year]):
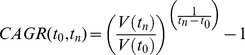



V(t_0_) – start value; V(t_n_) – end value; t_n_ – t_0_ : number of years.

#### 2. PARTAKE survey methodology

The PARTAKE questionnaire was developed to obtain information on clinical research awareness, perceptions, and expectations relevant to development of educational programs. Validity of the factors used in the survey was ascertained through review of the literature [10], [22], [23], [27], [28], [30]–[32], [36]–[38] and consultations with experts and activists representing all relevant stakeholders. The factors identified and included in the survey are:

- Knowledge of clinical research.
- Willingness to participate (WTP) in clinical trials.
- Trust in the clinical research establishment (including regulatory, industry, academia, and healthcare entities).
- Confidentiality.
- Compensation (including compensation for adverse outcomes).
- Altruism.
- Opinion and influence by familiar and respected individuals.
- Safety of research interventions and procedures.
- Access to sufficient information and clarifications about the research (transparency).
- Collaboration with foreign clinical research entities (industry and academia).

The PARTAKE Survey instrument is a 40-item, multiple-choice and open-ended questionnaire. It includes validation questions (mixed use of ‘True/False’ statements). The questionnaire was translated to Hindi and back-translated. The back-translation was determined to be equivalent to the original version by independent observers fluent in Hindi. During the development stage the questionnaire was tested on colleagues and co-workers from all social classes in English and Hindi. Each administration was followed by discussion and harmonization training. Guidelines were written into the questionnaire (Appendix S1) to ensure uniformity of administration.

#### 3. Ethics statement

The survey was approved by Institutional Ethics Committees of Medanta, The Medicity and Maulana Azad Medical College in Gurgaon and New Delhi, respectively. All participants were read the following statement: “You are about to participate in an anonymous survey of the knowledge and perception of clinical research in the general public. The survey should take about 15 minutes to complete. The purpose of the survey is to gain understanding of public awareness and knowledge of clinical research so that educational programs can be prepared to adequately inform the public about clinical research. The ultimate goal is to make the public an informed participant and partner in clinical research. The survey has been approved by the ethics committee of Medanta – The Medicity and ethics committee of Maulana Azad Medical College. Should you have any questions an experienced research professional would be available to answer them”. Survey data were collected only from those who provided verbal informed consent and agreed to proceed. Written consent was not obtained since the survey was anonymous to enhance accessibility to as wide a representation of the public as possible. For this reason identifying information (e.g., name, signature) was not collected. This verbal consent method was approved by the ethics committees. All exchanges with participants were recorded by the person administering the survey and/or one of the observers (all surveys were administered by at least 2 individuals, with one reading and collecting answers to survey questions and the other collecting any related exchanges not specifically noted in the survey).

#### 4. Statistical analyses

*Survey Administration Locations (* *Table 1* *).* Frequency distribution of locations in which the survey was administered. *Perceptions of clinical research (* *Table 2* *).* Responses were expressed in terms of percentages as ‘True’, ‘False’ and ‘Not Aware’. *Significant socioeconomic associations (* *Table 3* *).* In order to test whether perceptions of clinical research vary across socio-economic status of respondents such as education, income, and occupation, the p value for differences were calculated using standard normal deviate test (Z test). Only the significant results were reported in this exploratory analysis. *Differences in perceptions amongst those who ‘Heard’ and those who ‘Did Not Hear’ of clinical research (* *Table 4* *).* In order to identify significant differences in perceptions amongst these two groups the p value was computed using standard normal deviate test (Z test). Only significant results were reported in this exploratory analysis. SAS software (version 9.3) was used to analyze the data [39].

**Table 1:** Sites of PARTAKE survey administration in NCR (National Capital Region: New Delhi and Gurgaon).

Location	Population	Percent of Total
Botanical Gardens – Gurgaon	Mixed middle- and upper-class	19%
District Court – Gurgaon	Mixed low- middle- and upper-class	17%
Delhi Metro (transportation)	Mixed working class	6%
Jama Masjid - mosque	Mixed low- and middle class	6%
Jamia Hamdard – public university	College students	4%
GB Pant Hospital – Delhi center	Community hospital – staff, visitors, relatives of patients	21%
Chandni Chowk – Delhi center	Commercial, workers, mixed lower class	18%
Sangam Vihar	Lower class	9%

**Table 2:** Perceptions of clinical research.

		True (%)	False (%)	Not Aware (%)	N
	**General**				
1	Clinical research benefits society	94.1	3.5	2.3	170
2	Clinical research harms society	7.0	81.1	11.7	170
3	The most important reason for developing new treatments is the advancement of science	90.0	3.5	6.4	164
4	Clinical research is an essential step in developing new treatments	93.5	1.1	5.2	171
5	Hospitals that participate in clinical research provide better healthcare	67.2	8.7	23.9	171
6	The most important reason for developing new treatments is financial gain	47.9	44.4	7.6	171
	**Trust in Clinical Research**				
7	The government always adequately protects the public against unethical clinical research	56.7	26.3	16.9	171
8	Clinical research information provided by pharmaceutical companies can be trusted	56.1	26.3	17.5	171
9	Clinical research information provided by academic institutions can be trusted	81.2	8.7	9.9	171
10	If you decide not to participate in research your doctor will not give you good care	26.7	63.6	9.5	168
	**Ethics in Clinical Research**				
11	Doctors force their patients to participate in research	4.8	82.2	12.9	62*
12	Human participants in clinical research are treated like experimental animals (‘human Guinea Pigs’)	20.7	53.2	26.0	169
13	Participation in research is entirely voluntary	85.3	5.8	8.7	171
14	Volunteers in clinical research get adequate compensation for their participation	24.7	12.9	62.3	170
15	Participants in clinical research get adequate compensation for any adverse outcomes	28.6	21.6	49.7	171
16	Confidentiality is a matter of importance to research participants	71.1	22.9	5.8	170
17	Confidentiality of research participants is adequately protected	54.1	12.3	33.5	170
18	All the results of clinical research are made available to the public	37.0	33.8	29.0	62*
19	Altruism is the only valid reason for participation in research	62.5	25.1	12.2	171
20	Volunteers in clinical research get adequate information about the research they participate in	63.1	9.9	26.9	171

N – sample size; * – these items were late additions to the survey hence the smaller sample size.

**Table 3:** Significant socioeconomic associations.

Heard of Clinical Research						
Level	N (Sample Size)		Percentage		Z value	P value
Average	175		26.3			
Housewife	11		0		1.96	0.05
Income >1,000,000 INR	11		54		1.98	0.0478
**Human participants in clinical research are treated like experimental animals (‘human Guinea Pigs’)**						
**Level**	**N (Sample Size)**	**Proportion**		**Z value**		**P value**
Average	175	20.0				
Post Graduate	38	44		3.12		<0.01
Income >1,000,000 INR	11	63		3.31		<0.01

**Table 4:** Differences in perceptions amongst those who ‘Heard’ of clinical research and those who did not.

Human participants in clinical research are treated like experimental animals (‘human Guinea Pigs’)								
	Heard		Not Heard		Average		Z value	P value
	N	%	N	%	N	%		
True	16	33.3	20	16.0	36	20.8	2.51	0.012
False	22	45.8	68	54.4	90	52.0		
N/A	10	20.8	37	29.6	47	27.2		
Total	48	100	125	100	173	100		
**Altruism is the only valid reason for participation in research**								
	**Heard**		**Not Heard**		**Average**		**Z value**	**P value**
	**N**	**%**	**N**	**%**	**N**	**%**		
Yes	21	43.8	86	68.8	107	61.9	−3.03	<0.01
No	22	45.8	21	16.8	43	24.9		
NR	5	10.4	18	14.4	23	13.3		
Total	48	100	125	100	173	100		
**Participation in research is entirely voluntary**								
	**Heard**		**Not Heard**		**Average**		**Z value**	**P value**
	**N**	**%**	**N**	**%**	**N**	**%**		
Yes	36	75.0	110	88.0	146	84.4	−2.10	0.0358
No	7	14.6	3	2.4	10	5.8		
NR	5	10.4	12	9.6	17	9.4		
Total	48	100	125	100	173	100		

#### 5. Survey administration

The questionnaires were administered in person between March and May 2012. Participants were selected in public settings through pseudo-random, consecutive, convenience sampling. Inclusion criteria were: age 18 and above and no current participation in research. Field notes were made by the administrator and observers. At the end of each day the team gathered to discuss observations to improve quality of administration.

## Results

175 questionnaires were administered (109 in Hindi, 66 in English; Appendix S1).

### 1. Demographics

- Locations **(** **Table 1** **):** The survey was administered in 8 public locations, representing a broad cross-section of the population in the National Capital Region (NCR) around New Delhi, India.
- Age: 18–84 years; Mean: 39.6 (SD±16.6); Median: 36.5.
- Gender**:** 24% female.
- Literacy**:** Reading ability (81.7%);
- Writing ability (79.2%); Illiterate (7.3%).
- Income **(**INR/annum; [INR = Indian Rupees; exchange rate at the time of survey administration ∼50 INR = 1 USD]): 6.9% >1,000,000 INR; 20.0% 200,000–1,000,000 INR; 28.6% 50,000–200,000 INR; 19.4% Less than 50,000 INR; 25.1% did not report income.
- Education**:** 21.1% post-graduate, 32.0% College graduates, 24.0% Secondary, 11.4% Primary, and 11.4% provided no Information.
- Employment: 68.6% Employed, 13.1% Unemployed, 6.9% retired, 5.7% housewives. Data were not available for the remainder 5.7% of respondents.

### 2. Clinical Research Knowledge and Perceptions (Table 2, Figure 2)

**Figure 2:**
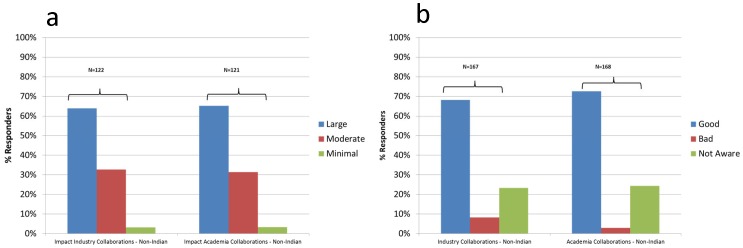
Industry and academic collaborations with non-Indian partners. Figure 2a) Impact. Question: What is the impact on clinical research for collaborations with non-Indian INDUSTRY/ACADEMIA partners? Answers: Good – Bad – None – Not aware Figure 2b) Magnitude. Question: What is the impact on clinical research for collaborations with non-Indian INDUSTRY/ACADEMIA partners? The extent of the impact: large, moderate or minimal.

Of respondents 26.3% reported having heard and 72.6% reported having not heard of clinical research. 2.9% stated they had participated in clinical research, 8.6% knew someone who did, and 58.9% expressed willingness to participate. Table 2 summarizes perceptions (‘True’, ‘False’, or ‘Not Aware’) in ‘General’, ‘Trust’, and ‘Ethics’ categories, and figure 2 foreign collaborations (Figure 2a ‘impact’ and Figure 2b ‘magnitude’ of collaboration impact).

### 3. Differences by socioeconomic groups (Table 3)

Exploratory analyses of differences amongst the various demographic groups were unremarkable except for the following significant associations: ‘heard of clinical research’ (lower in housewives and higher in the highest income group) and ‘Human participants in clinical research are treated like experimental animals (‘human Guinea Pigs’)’ (higher in post graduates and highest income group).

### 4. Differences in Perceptions amongst those who ‘Heard’ of Clinical Research and those who did not (Table 4)

An analysis of the differences in perceptions between those who responded ‘yes’ and those who responded ‘no’ to the question: “have you heard about clinical research?” – revealed non-significant differences in all items except: ‘Human participants in clinical research are treated like experimental animals (‘human Guinea Pigs’)’ (higher in those who ‘heard), ‘Altruism is the only valid reason for participation in research’ (lower in those who ‘heard’), and ‘Participation in research is entirely voluntary’ (lower in those who ‘heard).

## Discussion

The purpose of the ‘PARTAKE Survey of Public Knowledge and Perceptions of Clinical Research’ was to inform educational programs aimed at enhancing public knowledge, awareness, participation, and partnership in clinical research. Similar surveys have allowed identification of concerns and prejudice regarding participation in clinical research and enabled more appropriate decisions and involvement of patients and the public in clinical research [10], [18], [19], [40]. Interviewee demographics suggest a balanced representation of Indian urban population, though the 24% participation of females likely represents the difficulties approaching women in public areas and the percentage of illiterate interviewees (7%) is somewhat lower than the local illiteracy rate (at 14%; but similar to the 9% figure for males [41]). Validity of the survey was confirmed by consistent responses to opposing statements.

### 1. Positive Perceptions of Clinical Research

Overall, our results show that the majority of survey participants had a positive view of clinical research and of humans being part of it. An overwhelming majority endorsed research goals and benefits (e.g., 94.1% stated that research benefits society). The majority of responders expressed trust in the way clinical research is conducted. They were supportive of collaborations with international academic and pharmaceutical research partners but were more trusting of information provided by academic institutions than pharmaceutical companies. In addition, a majority (58.9%) indicated willingness to participate in some type of research. A 2005 review of several large US surveys concluded that the public has high regard for and considers health-related research a national priority [42]. Respondents in one of the surveys indicated that clinical research is of great value (68%), and only 2% thought there was no value [38]. This is similar to our findings (‘Research benefits society’ (‘False’ = 3.5%), ‘Research harms society’ (‘True’ = 7.0%)).

### 2. Negative Perceptions

A significant minority reported negative perceptions: e.g., ‘government always protects the public against unethical clinical research’ (False: 26.3%), ‘information provided by pharmaceutical companies can be trusted’ (False: 26.3%), ‘if you do not participate in research your doctor will not give you good care’ (26.7%), ‘participants are treated like guinea pigs’ (20.7%), ‘all clinical research results are made available to the public’ (False: 33.8%).

### 3. Knowledge of Clinical Research

There were several domains where a majority or a significant minority expressed lack of knowledge: of clinical research in general and of specific domains (e.g., compensation, ethical treatment of participants, confidentiality and availability of research data in the public domain).

Those who stated they have not heard of clinical research had very similar perceptions to those that had heard of clinical research but had a somewhat more positive view of ethics related issues concerning the participation of research volunteers (altruism, ‘guinea pig’ perception, and voluntariness of participation) (Table 4). Considering that the public’s main source of information about clinical research is the media and that reports in the media are often negative [26]–[29] our observation of significantly greater negative perceptions amongst those who ‘heard of clinical research’ is not surprising.

### 4. Participation and Willingness to Participate

In our survey 2.9% have indicated that they have previously participated in research while in the US 10% of adult interviewees stated they had ever participated in clinical trials, in Europe the average was 6%, and in India 8% [36], [37]. In our survey 58.9% demonstrated willingness to participate. Similarly, 54% of Americans have shown willingness to participate [38]. Another study found that 30.4% of public survey respondents expressed willingness to participate in clinical research [19]. Harris Interactive reported differences in factors affecting willingness to participate between the US and India with 72% of US respondents stating as ‘very likely’ the likelihood of participating in clinical research if they had a terminal illness versus 40% of Indians [37]. Clearly, more information about differences in the willingness to participate in clinical research needs to be explored.

### 5. Altruism

In our study the majority (62.5%) stated that altruism should be the main reason to participate in research. Among those who participated in clinical research 51% of responders from the US and 46% of responders from non-US countries (Europe and India) identified advancement of medicine and science as the main reason for their participation [33], [36], [37]. In the same studies 39% and 35%, respectively, indicated the main reason for participation was to help others with the condition. Altruism was independently associated with willingness to participate and adherence to medical regimen in a clinical trial [43]. In the same study 45.7% of participants reported one altruistic reason for participation and 20.6% altruism as the sole reason for participation.

### 6. Trust

The majority of participants in our survey trusted academia (81.2%), their physicians (63.6%), government (56.7%), and pharmaceutical companies (56.1%) to protect their rights. A US study found similar trends: personal physician (95% of respondents), academic institutions (93%), government (83%), media (55%), and pharmaceutical companies (53%) [38]. In the Kaiser Foundation study 27% did not think pharmaceutical companies can be trusted to provide accurate information on the safety of their drugs, similar to our finding of 26.3% of interviewees having lack of trust in ‘information provided by pharmaceutical companies’. Other studies reported negative association between lack of trust and willingness to participate in clinical trials [44]–[47].

### 7. Quality of Healthcare

Our data indicated that 67.2% believed ‘research hospitals provide better care’. In a US survey 82% associated research with ‘good hospitals’ [38].

### 8. Guinea Pig Metaphor

In our survey 20.7% thought that humans in clinical research are being used as ‘guinea pigs’. The Oxford Dictionary defines the ‘guinea pig’ metaphor as: “a person or thing used as a subject for experiment” [48]. The term may be used to indicate lack of autonomy (i.e., without consent), and/or without expected beneficence or concern for one’s safety [49], [50]. As such, it may imply an exploited vulnerability. Other studies have identified concerns about exploitation amongst patients and the public and the common use of the ‘guinea-pig’ metaphor to describe such maltreatment [44], [45], [51].

### 9. Ethical Concerns Regarding Conduct of Clinical Trials

Several questions in the survey addressed ethical matters relevant to the conduct of clinical research: coercion, protection by the government, confidentiality, autonomy of participants, ensuring that participants are informed about clinical research they participate in, and compensation (for participation and adverse outcomes). The majority of those surveyed expressed their belief that ethical principles are enforced, but significant minority either thought such principles were not enforced or was not aware of their proper enforcement. These matters received considerable attention, often negative, in both professional and lay media [12], [13].

### 10. Limitations of the Study

The sample was limited to those that were in urban public locations in the National Capital Region (NCR) of India. Limitations also include the small sample (175) and the population sampled being limited to a pseudo-random selection of Hindi- or English-speaking Indians that agreed to be interviewed.

### Conclusions

The purpose of the PARTAKE Survey was to study knowledge and perceptions of the public about clinical research in India. Our results demonstrate that the majority of survey participants had a positive view of clinical research and of human participation in it. An overwhelming majority endorsed research goals and benefits and a majority expressed trust in the way clinical research is conducted. Responders were supportive of collaborations with international academic and pharmaceutical research partners but were more trusting of information provided by academic institutions than pharmaceutical companies. The results suggest the majority of the public is aware of some key features of clinical research (e.g., purpose, value, voluntary nature of participation), and supports clinical research in general but a significant minority is unaware of other key features (e.g., compensation, confidentiality, protection of human participants, and availability of research data in the public domain) and exhibits some distrust in the conduct and reporting of clinical trials. Research guidelines and policies should be informed by results of such surveys. Public awareness and educational programs should be created to address negative perceptions and knowledge lacunae. Larger and cross-cultural surveys are required for generalization and guidance of such initiatives.

## Supporting Information

Appendix S1 PARTAKE Survey.
(DOCX)
