# Supplementary material for: PARTAKE Survey of Public Knowledge and Perceptions of Clinical Research in India
Source: PLoS One. 2013 Jul 16;8(7):e68666. doi: 10.1371/journal.pone.0068666 (PMC3713007; doi:10.1371/journal.pone.0068666)
Supplement: Appendix S1 — PARTAKE Survey. (DOCX) [file pone.0068666.s001.docx]

**APPENDIX S1: Survey of Public Knowledge and Perception of Clinical Research in India**

***Date:*** *______________* ***Interviewer:* __________________________ Start Time: ______ End Time: _____**

***Location:***  *____________________________________________* ***Age:*** *______* ***Gender:*** *M / F*

**Literacy:** Reading ability; Writing ability; Illiterate **Education:** primary, secondary, college, post-graduate

**Economic status:** employed / unemployed / retired / housewife

**Last 12-month income (in Indian Rupees):** 0 – 50,000; 50,000 – 2,00,000; 2,00,000 – 10,00,000; >10,00,000

*[Read the following to the individual surveyed*]: “You are about to participate in an anonymous survey of the knowledge and perception of clinical research in the general public. The survey should take about 15 minutes to complete. The purpose of the survey is to gain understanding of public awareness and knowledge of clinical research so that educational programs can be prepared to adequately inform the public about clinical research. The ultimate goal is to make the public an informed participant and partner in clinical research. The survey has been approved by the ethics committee of Medanta – The Medicity and ethics committee of Maulana Azad Medical College. Should you have any questions an experienced research professional would be available to answer them”.

1. **Have you heard about clinical research?** *Yes / No*

*[If ‘Yes’]:*

1. What was the source: doctor/media/internet/relatives/friends/colleagues/other:
2. Have you ever participated in clinical research? Yes / No
3. Do you know what clinical research is? Yes / No

*[A standard definition of clinical research is read to the person and opportunity is provided to ask questions and/or contact a clinical research professional for further clarifications]*

**Definition:** clinical research is a scientific method of studying the effects, both positive and negative, of proposed new treatments [medications or devices] in human volunteers (healthy individuals or patients). Government authorities require convincing demonstration of benefit of a new treatment before giving approval to use it in the public at large. If adverse effects are found with a new treatment then the benefits should outweigh the risk of adverse effects. Clinical trial, clinical study, and clinical research are all similar terms. Every medication in every pharmacy had to go through the clinical research process. Do you have any questions?

1. **Do you know anyone who participated in clinical research?** *Yes / No [If yes]:* How many?
2. **Do you know individuals who believe they were coerced to participate in clinical research?** *Yes / No*

*[If ‘Yes’]:* Who were they coerced by: Doctor/recruitment team/relatives/friends/not aware

1. **Will you be willing to participate in clinical research?** (Answer ‘Yes’ if there is any reasonable circumstance under which you see yourself participating) *Yes / No*

***[IF YES]:* What kind of involvement in clinical research would you be willing to undertake?** (Choose all appropriate answers; choose an answer if there is any reasonable circumstance where you see yourself accepting the option)

1. Single questionnaire (up to 20 minutes in duration)
2. Single blood draw
3. Single visit (up to a few hours in duration with multiple interventions)
4. Multiple visits (each up to a few hours in duration)
5. Multiple days of stay in a confined unit (including overnight stay)
6. Research done together with administration of standard medical care
7. Other: __________________________________________________________________________

***[IF NO]:* Would you like to share any specific reason/s?**

__________________________________________________________________________________________

1. **Would you have to take permission from someone else in order to participate in research?** *Yes / No*

**If so – who: spouse, parents, children, friends, physician, others: ________________________________________________________________________________________**

1. **Minimal compensation appropriate for one-day’s participation in a clinical trial (no overnight stay) is:**
2. Altruism
3. The worth of one day’s work plus expenses
4. Less than the worth of one day’s work plus expenses
5. More than the worth of one day’s work plus expenses
6. Just expenses (e.g., travel, parking expenses)
7. Free medical care
8. Depending on expected benefit (less benefit = more compensation)
9. Other: __________________________________________________________________________
10. **Please indicate if you believe the following statements are true, false, ‘not aware’, or ‘not relevant’:**
11. If you decide not to participate in research **your doctor will** **not give you good care**
12. **Confidentiality** is a matter of **importance** to research participants
13. **Confidentiality** of research participants is **adequately protected**
14. Clinical research information provided by **pharmaceutical companies** can be trusted
15. Clinical research information provided by **academic institutions** can be trusted
16. The most important reason for developing new treatments is **financial gain**
17. Clinical research **benefits** society
18. Volunteers in clinical research get **adequate compensation** for their participation
19. Clinical research is an essential step in **developing new treatments**
20. Human participants in clinical research are **treated like experimental animals (‘human Guinea Pigs’)**
21. The most important reason for developing new treatments is the **advancement of science**
22. **Altruism** is the only valid reason for participation in research
23. Volunteers in clinical research **get adequate information** about the research they participate in
24. Participation in research is entirely **voluntary**
25. Participants in clinical research get **adequate compensation** for any adverse outcomes
26. Clinical research **harms** society
27. **The government always** adequately protects the public against unethical clinical research
28. Hospitals that participate in clinical research **provide better healthcare**
29. All the results of clinical research are made **available to the public**
30. Doctors **force their patients** to participate in research
31. **Other beliefs or statement you wish to make regarding clinical research:**

__________________________________________________________________________________________

1. **What is the impact on clinical research for collaborations with non-Indian INDUSTRY partners?**

Good – Bad – None – Not aware **The extent of the impact:** large, moderate or minimal

1. **What is the impact on clinical research for collaborations with non-Indian ACADEMIC partners?**

Good – Bad – None – Not aware **The extent of the impact:** large, moderate or minimal

Are there any other comments you would like to bring to our attention:

__________________________________________________________________________________________
